# The Protein Data Bank: Current Status and Future Challenges

**DOI:** 10.6028/jres.101.025

**Published:** 1996

**Authors:** Enrique E. Abola, Nancy O. Manning, Jaime Prilusky, David R. Stampf, Joel L. Sussman

**Affiliations:** Department of Chemistry, Brookhaven National Laboratory, Upton, NY 11973 USA; BioInformatics Unit, Weizmann Institute of Science, Rehovot 76100 Israel; Department of Chemistry, Brookhaven National Laboratory, Upton, NY 11973 USA; BioInformatics Unit, and the Department of Structural Biology, Weizmann Institute of Science, Rehovot 76100 Israel; Departments of Biology and Chemistry, Brookhaven National Laboratory, Upton, NY 11973 USA

**Keywords:** database, federation, NMR, protein structure, three-dimensional structure, x-ray crystallography

## Abstract

The Protein Data Bank (PDB) is an archive of experimentally determined three-dimensional structures of proteins, nucleic acids, and other biological macromolecules with a 25 year history of service to a global community. PDB is being replaced by 3DB, the Three-Dimensional Database of Biomolecular Structures that will continue to operate from Brookhaven National Laboratory. 3DB will be a highly sophisticated knowledge-based system for archiving and accessing structural information that combines the advantages of object oriented and relational database systems. 3DB will operate as a direct-deposition archive that will also accept third-party supplied annotations. Conversion of PDB to 3DB will be evolutionary, providing a high degree of compatibility with existing software.

## 1. Introduction

The Protein Data Bank (PDB) is an archive of experimentally determined three-dimensional structures of proteins, nucleic acids, and other biological macromolecules [1, 2]. PDB has a 25 year history of service to a global community of researchers, educators, and students in a variety of scientific disciplines [3]. The common interest shared by this community is a need to access information that can relate the biological functions of macromolecules to their three-dimensional structure. PDB is now being replaced by the 3DB, Three-Dimensional Database of Biomolecular Structures, which will continue to operate from Brookhaven National Laboratory.

The challenge facing the new 3DB is to keep abreast of the increasing flow of data, to maintain the archives as error-free as possible, and to organize and present this information in ways that facilitate data retrieval, knowledge exploration, and hypothesis testing without interrupting current services. The PDB introduced substantial enhancements to both data management and archive access in the past two years, and is well on the way to converting to a more powerful system that combines the advantages of object oriented and relational database systems. 3DB will transform PDB from a data bank serving solely as a data repository into a highly sophisticated knowledge-based system for archiving and accessing structural information. The process will be evolutionary, insulating users from drastic changes and providing both a high degree of compatibility with existing software and a consistent user interface for casual browsers.

Development is under way for 3DB to operate as a direct-deposition archive. Mechanisms are provided for depositors to submit data with minimal staff intervention. Data archived in 3DB is managed using the Relational Database Management System (RDBMS) from SYBASE^1^ [4]. The new database (3DBase) is being developed with a view towards being a member of a federation of biological databases. Collaborative international centers are also being established to assist in data deposition, archiving, and distribution activities.

## 2. Resource Status—1995

Rapid developments in preparation of crystals of macromolecules and in experimental techniques for structure analysis have led to a revolution in structural biology. These factors have contributed significantly to an enormous increase in the number of laboratories performing structural studies of macromolecules to atomic resolution. Advances include: 1) recombinant DNA techniques that permit almost any protein or nucleic acid to be produced in large amounts; 2) faster and better x-ray detectors; 3) real-time interactive computer graphics systems, together with automated methods for structure determination and refinement; 4) synchrotron radiation, allowing the use of extremely tiny crystals, Multiple Wavelength Anomalous Dispersion (MAD) phasing, and time-resolved studies via Laue techniques; 5) NMR methods permitting structure determination of macromolecules solution; and 6) electron microscopy (EM) techniques, for obtaining high-resolution structures of two-dimensional crystals.

**Fig. 1:**
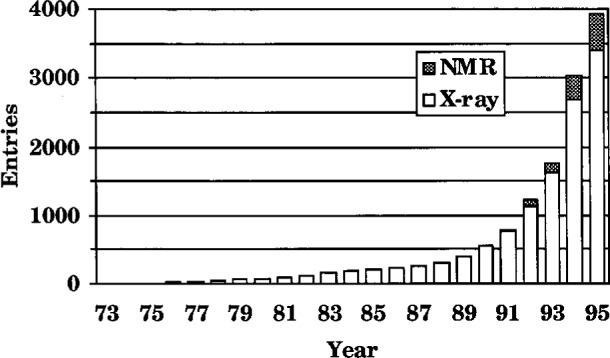
Yearly depositions to PDB.

These dramatic advances produced an abrupt transition from the linear growth of 15–25 new structures deposited per year in the PDB before 1987 to a rapid exponential growth reaching the current rate of approximately 25 deposits per week (Fig. 1). This rapid increase overwhelmed PDB staff resources and data processing procedures and, by mid-1993, a backlog of some 800 coordinate entries had accumulated. By January 1994 this backlog was eliminated by increased automation of processing and the addition of new staff. In all, more than 3000 of the nearly 4000 current PDB coordinate entries (approximately 75 %) have been processed since 1991. Table 1 is a summary of the contents of PDB. Present staff now keep abreast of the deposition rate with a timeline of three months from receipt to final archiving, which includes the time that the entry is with the depositor for checking. This timeline is comparable to the publication schedules of the fastest scientific journals.

**Fig. 2:**
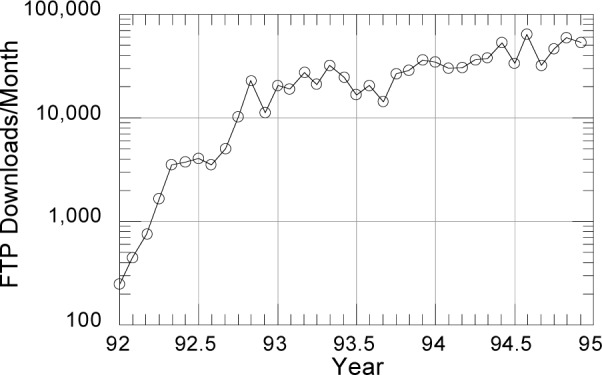
FTP accesses to PTB.

In the same period, the proliferation and increasing power of computers, the introduction of relatively inexpensive interactive graphics, and the growth of computer networks greatly increased the demand for access to PDB data (Fig. 2). The requirements of molecular biologists, drug designers, and others in academia and industry were often fundamentally different from those of crystallographers and computational chemists, who had been the major users of the PDB since the 1970s.

PDB entries are accessible by FTP, the World Wide Web (WWW), and on CD-ROM. PC users of the CD-ROM are provided with the browser, PDB-SHELL [5], built using the FoxPro^®^ RDBMS [6]. In addition to its browsing mechanisms, PDB-SHELL provides direct access to the public-domain molecular viewing program RasMol [7]. Recent enhancements to PDB’s WWW server (http://www.pdb.bnl.gov) have greatly improved the accessibility and utility of the archive over the Internet. This includes the release of PDBBrowse [8, 9], which is accessible through the World Wide Web.

PDBBrowse incorporates a number of features that make it easy to access information found in PDB entries. Multiple search strings covering various fields corresponding to PDB record types such as compound, header, author, biological source, and heterogen data, are supported. These searches support Boolean “and”, “or”, and “not” operators. Entries selected can be retrieved automatically, and the molecular structures can be displayed using RasMol or other viewers. Entries include links to information resources such as SWISS-PROT [10], BMRB [11], the Enzyme Commission Database [12], and the Entrez Reference Database [13].

Internet access to the archives has become the primary mode of retrieving entries from the PDB. However, we continue to receive a considerable number of orders for the CD-ROM product. We anticipate that this will continue to be true for a variety of reasons. For example, network performance still remains poor in a number of locations, and these disks, released quarterly, provide local access to the contents of the archives. Some of these network access difficulties may be easily overcome by installing a copy of the PDB FTP and WWW servers using mirroring software. With this software all files in the PDB are stored locally and changes are automatically reflected on a daily basis.

## 3. The 3-Dimensional Database of Biomolecular Structures—3DB

**Fig. 3:**
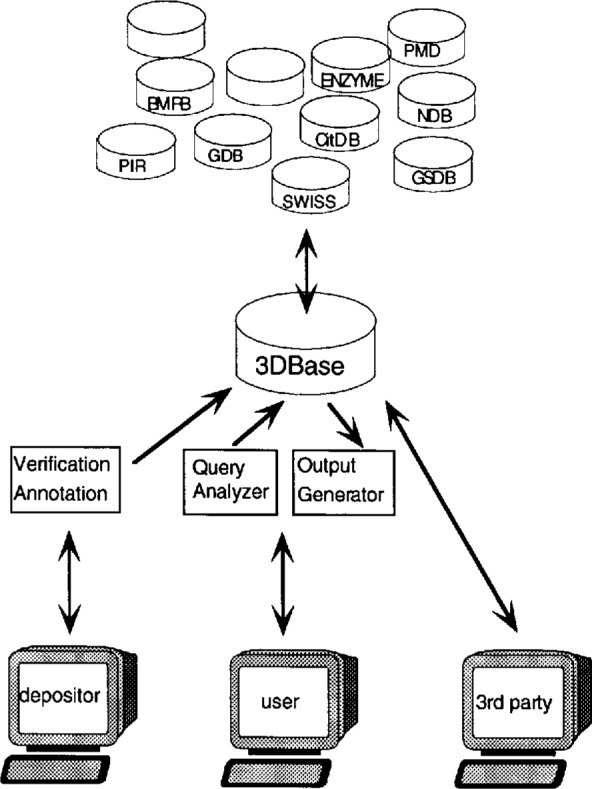
3DBase.

Converting PDB to 3DB entails changes to every aspect of current operations. A new data submission and archival system is being designed which attempts to balance the need for full automation with the need to maintain very high levels of data accuracy and reliability. The new system relies on an RDBMS for data management. An overview of the relationships between 3DBase and depositors, users, third party software developers, and other databases is shown in Fig. 3. The following sections give a summary of development work.

### 3.1 The 3DBase—A Relational Database Management System for 3DB

3DBase is constructed with the SYBASE RDBMS, the Object-Protocol Model (OPM), and the OPM data management tools [14] developed by Dr. Victor Markowitz’s group at Lawrence Berkeley National Laboratory. SYBASE provides a powerful and robust environment for data management; the OPM tools allow rapid development of SYBASE databases; and OPM’s object-oriented view provides a scientifically intuitive representation of data. Along with a graphical schema editor, Markowitz’s group distributes a number of other development tools; foremost is a schema translator that generates SQL statements for building tables, indices, and constraint rules and triggers.

This development effort attempts to address the needs of the diverse user community served by the PDB. The schema supports queries related to crystallographic as well as molecular biology questions. The database is being designed with the idea that in the near future it will be federated with other biological databases. Our expectation is that through federation, complex queries may be submitted to our database for which answers that originate from several databases may be easily returned. Interoperability is addressed through the use of schema sharing with other OPM-based databases and support for a variety of data interchange formats in query results.

In addition to providing users with a powerful environment capable of complex ad hoc queries, 3DBase will also facilitate management of the growing archives, which are expected to contain over 30 000 structural reports by the year 2000.

This work is being done as a collaboration among the following groups:

- The Protein Data Bank—Brookhaven National Laboratory
- BioInformatics Unit—Weizmann Institute of Science
- OPM Data Management Tools Project—Lawrence Berkeley Laboratories
- The Genome Data Base - Johns Hopkins University

### 3.2 Schema Development

OPM is a semantic data model that includes constructs that are powerful enough to represent the diversity and complexity of data found in PDB entries. OPM has constructs such as object class, object attribute, class hierarchy and inheritance, and derived attribute. A schema for 3DBase has been developed using OPM and is available for perusal through the PDB WWW home page. Among its notable features is a description of the coordinate data set from two perspectives. The object class oExperiment provides users with the classical view of a PDB entry that is a report of crystallographic or NMR analysis. An alternative view is presented in the class oMacroMolecule that describes the biologically active form of the molecule. Appendix A provides a description of these object classes. An example that clearly demonstrates the differences between these classes is the case of the hemoglobin molecule. The oExperiment object contains the coordinates for the crystallographic asymmetric unit which in most cases is a dimer. The full tetramer will however be presented in the oMacroMolecule object. The latter case is normally what molecular biologists are interested in when accessing PDB entries. However, crystallographers wishing to do packing studies or further refinement will need access to the oExperiment object.

In 3DBase, literature citation data are being loaded into the CitDB database of references that was developed by GDB [15]. A pointer to the appropriate entry in CitDB is loaded in the oExperiment object of 3DBase. This is an example of the strategy that we are following in linking to external databases. CitDB will be managed as a federation of a number of database centers, including GDB and PDB. There are several advantages to this scenario. By sharing the schema and management of the citation database, access to information stored in each of the databases via the bibliographic citation becomes straightforward. Duplication of effort is also minimized. Today it is still common to have several public databases build and maintain their own bibliographic databases. This will no longer be economically feasible with the expected rapid growth in database size.

### 3.3 Building Semantic Links to External Data Sources

Links to contents of sequence databases are provided in 3DBase via the oPrimarySeq and oSeqAdv classes. These classes form another set of objects that link 3DBase objects to external databases. Representing, building, and maintaining these links will be one of 3DB’s primary tasks in the coming years. There are several issues that must be addressed for this effort to succeed. Data representation issues are foremost. Each database uses a different data model to represent and store information. Semantic contents are rarely the same, for example the primary sequence data stored in sequence databases such as SWISS-PROT and PIR [16, 17] are presented using a view which differs significantly from that used by PDB.

PIR and SWISS-PROT entries present information on the wild-type molecules. Each entry normally contains the sequence of one gene product and some entries include the complete precursor sequence. Annotation is provided to describe residue modifications. In both databases, the residue names used are limited to the twenty standard amino acids.

In contrast, PDB entries contain multichain molecules with sequences that may be wild type, variant, or synthetic. Sequences may also have been modified through protein engineering experiments. A number of PDB entries report structures of domains cleaved from larger molecules.

The oPrimarySeq object class was designed to account for these differences by providing explicit correlations between contiguous segments of sequences as given in PDB ATOM records and in the PIR or SWISS-PROT entries. Several cases are easily represented using this class. Molecules containing heteropolymers will be linked to different sequence database entries. In some cases, such as those PDB entries containing immunoglobulin Fab fragments, each PDB chain may be linked to several different SWISS-PROT entries. This facility is needed because these databases represent sequences for the various immunoglobulin domains as separate entries. oPrimarySeq is also able to represent molecules engineered by altering the gene, e.g., fusing genes, altering sequences, creating chimeras, or circularly permuting sequences. In addition, it will link segments of the structure to entries in motif databases (e.g., PROSITE [18], BLOCKS [19]).

Initial building of these links is straightforward and requires analysis of a few entries coming out of a FASTA [20] or BLAST [21] search against the sequence databases. What may be problematic in the long run is updating these links as new experimental evidence is encountered, leading to a correction in either database. Both PIR and SWISS-PROT have similar problems as they build pointers to PDB entries. To help obviate these difficulties we have agreed to establish a closer interaction between the databases. We are setting up a protocol that will broadcast to each database changes that occur which in turn could affect specific entries.

## 4. Data Deposition

3DB will operate as a direct-deposition archive, providing mechanisms that will allow depositors to load data with minimal staff intervention. This strategy is essential if 3DB is to meet present projections of exponential growth in depositions against a fixed staff size. This is particularly challenging due to the complexity of the data being handled, the need for a common viewpoint of the entry description, and the community requirement that these data be accessible immediately upon receipt.

With direct deposition, there will be a concomitant need to increase the power of data validation procedures. These procedures must reflect current models for identifying errors and must be as complete as possible. Quality control issues assume a more central and difficult role in direct deposition strategies. Distributed data must be of the highest quality; otherwise users will lose their trust in the archived data and will have to revalidate data received from 3DB before using them, clearly an unproductive scenario.

### 4.1 Current Data Deposition Procedures

Since its inception in 1971, the method followed by the PDB for entering and distributing information paralleled the review and edit mode used by scientific journals. Currently, the author submits information which is converted into a PDB entry and run against PDB validation programs by a PDB processor. The entry and the output of the validation suite are then evaluated by PDB scientific staff members, who complete the annotations and return the entry to the author for comment and approval. Table 2 summarizes checks included in our current data validation suite. Corrections from the author are incorporated into the entry, which is reanalyzed and validated before being archived and released.

Originally data flow was a manual system, designed for a staff of one-to-two scientists, and a deposition rate of 25–50 entries per year. One person processed an entry from submission through release. By the late 1980s, when the first steps at automation were being introduced, running the validation programs took about 4 hours per entry. Today, the same step, which includes a vastly improved set of validation programs, takes about one minute. Graphical viewing of data, a useful and powerful annotating and checking tool, has been available to processors since 1992.

The current deposition load of approximately 100 entries each month is handled by 10 staff members, who annotate and validate entries. The process is a production line in which checking is repeated at various steps to ensure that errors and inconsistencies in data representation are minimized. Prior to June 1994, a significant number of depositions required that administrative staff keyboard information provided in a deposition form. Introduction of the current Electronic Deposition Form and a new parsing program have eliminated this step for most submissions.

Today, most of the processing time is spent resolving data representation issues and ensuring that outliers are identified and annotated. The most troublesome areas consistently are those involving handling of heterogens, resolving crystal packing issues, representing molecules with non-crystallographic symmetry, and resolving conflicts between the submitted amino acid sequence and that found in the sequence databases. Publications and other references are sometimes consulted to verify factual information such as crystal data, biological details, reference information, etc. Processing programs, although much improved from those used in 1991, still allow errors to pass undetected through the system, requiring a visual check of all entries. We continually improve these programs and acquire software from collaborators to address deficiencies that both we and our users have identified. In addition, we now have formed a quality control group that will be looking into our operations to identify sources of errors and to recommend steps to improve data quality.

### 4.2 Development of Automatic Deposition and Validation

**Fig. 4:**
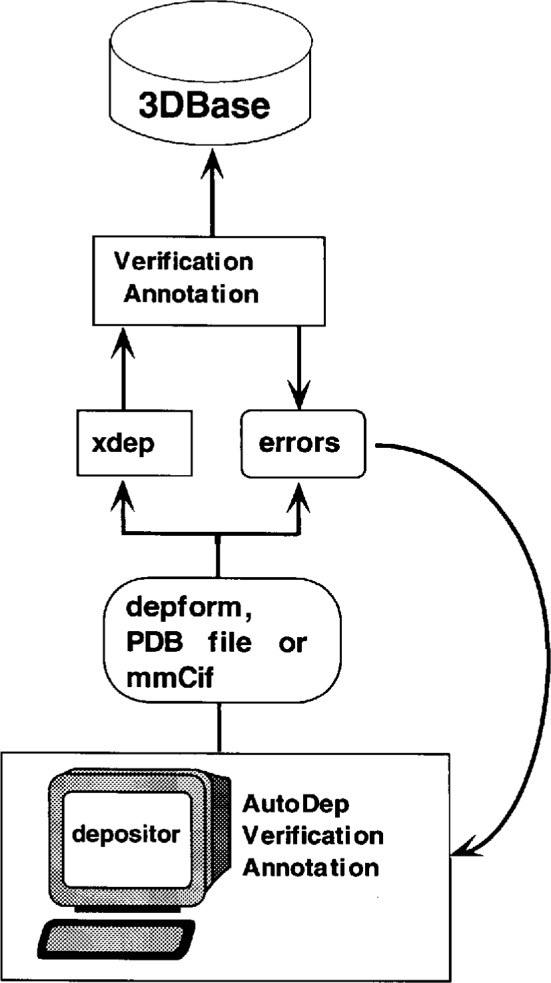
AutoDep protocol.

3DB must overcome many challenges for direct deposition to work. In a recent workshop held to assess the needs of 3DB users, crystallographers and NMR spectroscopists were unanimous in their desire to have a system that did not require additional work on their part when depositing data. On the other hand, consumers (which included these same depositors) were vocal in their desire for entries to contain more information than what is currently available within the PDB. We are striving to develop a suite of deposition and validation programs that accommodates these somewhat conflicting desires while ensuring that the archives maintain the highest standard of accuracy. A schematic diagram of the automatic deposition process is depicted in Fig. 4.

AutoDep, the new automatic deposition program, is designed to simplify the deposition process. It includes a convenient and interactive electronic deposition form that guides the author in providing information. It also contains tools for data verification and validation and is able to flag errors in syntax or spelling. A considerable variety of information, which must be supplied by the authors, is archived about each structure. The form requests the same information as the electronic deposition form, but helps ease the burden of filling it out by populating fields using data from existing PDB entries or other computer-generated output (e.g., X-PLOR output). These data can then be reviewed and modified.

Checks against other databases are an important and evolving part of this process. For example, names of organisms are checked against the taxonomy database of the National Center for Biotechnology Information (NCBI) [22], chemical names against IUPAC nomenclature tables [23], and author names and citations against MEDLINE [24] (CitDB when it becomes available). FASTA/BLAST programs are run against the SWISS-PROT and PIR databases to verify protein sequences, and variant and mutant sequences are checked against the Protein Mutant Database (PMD) [25]. Links between the PDB/3DB entry and these databases are established in the process. To handle the increasing number of entries with nonstandard residues (heterogens), a standard residue and heterogen dictionary is being developed to be used in the data entry and checking process. We are also adopting programs developed by the Cambridge Crystallographic Data Center (CCDC) [26] to handle heterogens automatically.

In addition to the deposition form that is filled out through AutoDep, authors must submit the coordinate file and other experimental data files for processing and archiving. Facilities are provided by AutoDep that help simplify this process. An FTP script is generated that takes author-specified local files and uploads these data to the PDB server.

The completed form is then converted automatically into a PDB formatted file and, along with the coordinate data, is submitted to a set of validation programs for checking and further annotation. These programs are designed to check: 1) the quality, consistency, and completeness of the experimental data; 2) possible violations of physical or stereochemical constraints (e.g., two atoms in the same place, appropriate bond angles, etc.); 3) compliance with our data dictionary (syntax checks); and 4) the correspondence of the experimental data to the derived structure (in the near future). Development of the validation suite will evolve with advice from the community and encompass programs currently in use, written both within and outside the PDB.

The validation software automatically generates and includes in the entry measures of data quality and consistency as well as annotations giving details of apparent inconsistencies and outliers from normal values. This output is returned to the depositor for review. Entries whose data quality and consistency meet appropriate standards may then be sent by the depositor directly for automatic entry into the database. Entries that do not pass the quality and consistency checks may be revised by the depositor to correct inadvertent errors. Alternatively, the depositor may decide to do more experimental work in order to resolve problems.

Apparent inconsistencies or outliers remaining in a submitted entry must be explained by the depositor in an annotation. In the most interesting cases, unusual features are a valid and important part of the structure. However, all such entries will be reviewed for possible errors by 3DB staff, who may discuss any important issues with the depositor. The 3DB staff will then forward acceptable entries to the database.

To make automatic deposition as easy as possible, we are working with developers of software commonly used by our depositors. By modifying these programs to produce compliant data files and performing validation and consistency checks before submission, it may be possible to bypass most of the tedious steps in deposition. We are already working with Dr. Axel Brünger to use procedures available through X-PLOR [27] to replace part of the validation suite for structures produced by x-ray crystallography and NMR. Diagnostic output will be included automatically as annotations in the entry. A limited version of X-PLOR will be available from 3DB to all depositors for validation purposes only.

Validation of coordinate data against experimental x-ray crystallographic data requires access to structure factor data, which are requested by PDB, the International Union of Crystallography (IUCr), and some journals but are not always supplied by the depositor. We are working toward building a consensus in the community that structure factor data are a necessary component of deposits of structures derived by x-ray crystallography. Statistics such as number of F’s and R-values vs. sin(theta)/lambda, will be calculated and included in the 3DB entry as annotations to the experiment.

In order to make it easier for depositors to submit structure factors (as well as to exchange these data between laboratories), the PDB, in close collaboration with a number of macromolecular crystallographers, has developed a standard interchange format for these data. This standard is in CIF and was chosen both for simplicity of design and for being clearly self-defining, i.e., that the file contains sufficient information to be read and understood by either a program or a person. Details of this format are available through the PDB WWW server.

A consensus is still developing in the NMR community as to what types of experimental data should be deposited and what kinds of validation and consistency checks should be performed. Structural data produced by other methods may also have special features that should be archived or checked. Requirements for the types of data to be deposited and proper ways of checking the validity and consistency of the data will be developed in cooperation with the experimental community for each type of structure data archived by the 3DB.

## 5. Accessing Data in 3DBase—User Queries and Report Generation

**Fig. 5:**
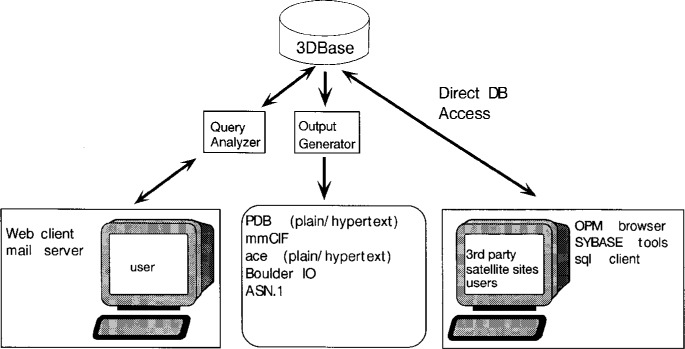
Access to 3DBase.

Primary access to 3DBase will be via the network using general purpose graphical user interfaces like Mosaic or Netscape. Access will also be available through the use of software developed by third parties (commercial developers). As diagrammed in Fig. 5, user queries will be addressed to the Query Analyzer (3DB-QA), a program module running at the server site that will parse queries and pass them on to 3DBase. Query results will be returned through the Output Generator (3DB-OG) in the format requested by the user.

Queries placed over the network will generally be in the form of URLs, which are easily generated from hypertext links, HTML-based forms, or by programs or scripts using the National Center for Supercomputing Applications libraries [28] for more sophisticated applications. As part of the query the user may specify the format of the response, as we do at the present time in the PDB WWW browser. The response frequently will be in the form of an HTML document, but it can also be a PDB- or CIF-formatted file [29, 30]. The information returned may be either a complete or partial entry, or information from linked databases or external programs.

A 3DBase browser has been built using Dr. Stan Letovsky’s Genera system [31]. Users specify search criteria by filling out an HTML form. Software at BNL processes this form and generates the required SQL. System performance is improved by using stored SYBASE SQL procedures that access each predefined object. The fields available are similar to those in our PDBBrowse program.

For those familiar with (or willing to learn about) the OPM protocol, access to the object layer will be provided using a high level OPM-based query language. As part of the 3DB open database policy, direct access to the underlying RDBMS will be allowed and actively supported. These queries are not parsed by the 3DB-QA module, so better response time can be expected. This provides third party developers with the opportunity to either incorporate SQL clients in their products or to learn more about the OPM protocol and thereby gain access to all of the benefits that the Object model affords, e.g., active external links, programs, etc.

As depicted in Fig. 5, the output generator will return query results using a variety of data interchange formats. PDB will continue to support its current format for the foreseeable future. We plan to extend this format to allow us to represent objects being stored in 3DBase. In addition, a “raw format” is being provided which returns an attribute/value pair. This form is easily parsed and is more compact than PDB format.

## Figures and Tables

**Table 1:** PDB holdings—November 1995

Total holdings	
3928	released atomic coordinate entries
576	structure factor files
109	NMR restraint files
**Molecule type**	
3570	proteins, peptides, and viruses
80	protein/nucleic acid complexes
266	nucleic acids
12	carbohydrates
**Experimental technique**	
125	theoretical modeling
521	NMR
3694	diffraction and other

**Table 2:** Data validation with current system

Class	What is checked
Stereochemistry	Bond distances and angles, dihedral angles (Ramachandran plot), planarity of groups, chirality
Bonded/non-bonded interactions	Crystal packing, unspecified inter- and intra-residue links
Crystallographic information	Matthews coefficient, *Z*-value, cell transformation matrices
Noncrystallographic transformations	Validity of noncrystallographic symmetry
Primary sequence data	Discrepancies with sequence databases
Secondary structure	Generated automatically or visually checked
Heterogen groups	Geometry and nomenclature
Miscellaneous checks	Solvent molecules outside the hydration sphere, syntax checks, internal data consistency checks

## Footnotes

1 Certain commercial equipment, instruments, or materials are identified in this paper to foster understanding. Such identification does not imply recommendation or endorsement by the National Institute of Standards and Technology, nor does it imply that the materials or equipment identified are necessarily the best available for the purpose.

## 6. Appendix A.

### Description of Two Primary Database Objects

The following examples describe two primary object classes found in 3DBase. OPM schema consists of definitions of object classes, each of which is described by a set of attributes. Data types assigned to attributes can be primitives such as numbers or character strings or they can be other classes defined in the schema. In addition to object classes, OPM provides controlled value classes that restrict the allowed values for objects in the class. Attributes can be either single- or multiple-valued. The latter can be specified as an ordered list (list-of) or as an unordered list (set-of).

Object classes are grouped into a hierarchy of subclass and superclass relationships, called an ISA hierarchy. A class is said to inherit all the attributes of its superclass. In the description below, object classes have been assigned names which start with the small letter “o”, attributes with the small letter “a”, and controlled value classes with the prefix “cv”.

#### oExperiment

isa o3DBExportObj

*aTitle: list-of varchar(255)
  required
  Description: Contains a title for the experiment or analysis that is represented in the object. It should identify an entry in the PDB in the same way that a title identifies a paper.
*aDepositor: list-of oPerson
  required
  Description: An ordered list of names with address information.
*aKeywords: set-of varchar(80)
  optional
  Description: Set of keywords relevant to the entry. These provide a simple means of categorizing the experiment or the molecules studied.
*aExpdta: cvExperimentTypes
  required
  Description: Identifies the experimental technique used in the study. This normally refers to the type of radiation and sample, but can also include the spectroscopic or modeling technique. Permitted values include:
ELECTRON DIFFRACTION
FIBER DIFFRACTION
FLUORESCENCE TRANSFER
NEUTRON DIFFRACTION
NMR
THEORETICAL MODEL
X-RAY DIFFRACTION
*aReference: list-of oExternalReference
  optional
  Description: Publications related to the study. These citations are chosen by the depositor.
*aRemarks: set-of oAnnotations
  optional
  Description: General comments regarding the experiment or the molecules studied.

#### oMacroMolecule

isa o3DBExportObj

*a3DBID: char(4)
  required
  Description: Contains the PDB identification code. This is a four-character field in which the first character must be an integer greater than zero. This identifier is unique within PDB and is randomly assigned to an entry.
*aMolName: set-of varchar(80)
  optional
  Description: Set of molecule names. Each molecule may be assigned more that one name allowing for the use of synonyms and aliases.
*aSrcDescr: oSource
  optional
  Description: Specifies the biological and/or chemical source of each biological molecule in the entry. Sources are described by both the common name and scientific names, genus, and species. Strain and/or cell-line for immortalized cells are given when they help to uniquely identify the biological entity studied.
*aMolType: cvMacroMoleculeType
  optional
  Description: Molecule type -Valid values are:
  protein
  DNA
  RNA
  polysaccharide
  other - must annotate
*aBioMol: varchar(255)
  optional
  Description: Information on accessing the structure of the complete biological molecule. Currently this contains the filename for a biomol entry found on the PDB FTP server. This attribute will be replaced by a new object class with attributes that provide the transformation matrices, descriptive text, and if available, the filename for the coordinate set.
*aCoordinates: set-of oChain
  optional
  Description: Atomic coordinate values stored as individual chains
*aMolSequence: list-of oPrimarySeq
  optional
  Description: SEQRES records contain the amino or nucleic acid sequence of residues in each chain of the macromolecule.
*aDomain: varchar(100)
  optional
  Description: Specifies a domain or region of the molecule.
*aEngineered: cvFlagDict
  optional
  *aEnzyme: set-of oExternalReference
  optional
  Description: The Enzyme Commission number associated with the molecule.
*aMutation: varchar(255)
  optional
  Description: Describes the mutations present.
*aFormula: varchar(80)
  optional
*aMolWeight: float
  optional
*aMolID: cvLocalID
  required
  Description: Integer to uniquely identify each instance of a coordinate set for a molecule. For example, each occurrence of lysozyme in the database will be identified by a unique number.
*aAnnotate (aSummLine, aExtDB): set-of (varchar(255), oExternalReference)
  optional
  Description: Annotations describing the molecule. This is presented as a table of text and pointers to external databases.

## 7. Appendix B

### 3DBase Report in Different Formats

Raw Format

For the oMacromolecule object of the entry 1ACE:

*Export_Object: 1ACE
*Macromol_name: Acetylcholinesterase
*Macromol_name: Ache
*EC_number: 3.1.1.7
*3DB_init_res_num: 4
*3DB_term_res_num: 534
*Init_res_num: 25
*Term_res_num: 555
*Databae_ID_code: ACES_TORCA
*Domain_desc: No
*Engineered: No
*Source_sci_name: Torpedo californica
*Source_common_name: Pacific electric eel

Boulder IO Format:

Export_Object = 1ACE
Macromol_name = Acetylcholinesterase
Macromol_name = Ache
EC_number = 3.1.1.7
Chain = {
  3DB_init_res_num = 4
  3DB_term_res_num = 534
  Init_res_num = 25
  Term_res_num = 555
  Database_ID_code = ACES_TORCA
  }
Domain_desc = No
Engineered = No
Source_sci_name = Torpedo californica
Source_common_name = Pacific electric eel
